# Evaluation and Comparison of Laboratory Methods in Diagnosing *Mycobacterium tuberculosis* and Nontuberculous Mycobacteria in 3012 Sputum Samples

**DOI:** 10.1111/crj.70071

**Published:** 2025-03-14

**Authors:** Qian Wu, Yelei Zhu, Yu Zhang, Zhengwei Liu, Mingwu Zhang, Jiazhen Chen, Beibei Wu

**Affiliations:** ^1^ Department of Infectious Diseases, Shanghai Key Laboratory of Infectious Diseases and Biosafety Emergency Response, National Medical Center for Infectious Diseases, Huashan Hospital, Shanghai Medical College Fudan University Shanghai China; ^2^ Zhejiang Key Lab of Vaccine, Infectious Disease Prevention and Control Zhejiang Provincial Center for Disease Control and Prevention Hangzhou China; ^3^ School of Public Health Hangzhou Medical College Hangzhou China; ^4^ School of Public Health Wenzhou Medical University Wenzhou China

**Keywords:** combined test sensitivity, diagnosis, mycobacteria, *Mycobacterium tuberculosis*, nontuberculous mycobacteria

## Abstract

Tuberculosis is a global public health threat as an infectious disease, and effective blocking of transmission relies on timely diagnosis. Although a number of laboratory tests are available in diagnosing *Mycobacterium tuberculosis* (MTB) and nontuberculous mycobacteria (NTM), it is still of great need to evaluate their diagnostic value in clinical samples. In this study, we evaluated five MTB diagnostic methods (including conventional sputum smear microscopy, sandwich cup sputum smear microscopy, sputum culture, Xpert‐MTB/RIF, and CapitalBio TB/NTM kit detection test) in 3012 sputum specimens and compared their diagnostic performance of the single and combined tests. In the diagnosis of MTB, the Xpert‐MTB/RIF had the highest sensitivity, 79.6% (0.770–0.819), among all the single diagnostic methods, and the combination of CapitalBio TB/NTM kit and culture approach significantly increased sensitivity to 88.4% (*p* < 0.05). In the diagnosis of NTM, the culture method has higher sensitivity (85.7%) compared with the Capital Bio TB/NTM kit method (45.7%). In the diagnosis of mycobacteria, the CapitalBio TB/NTM kit detection test has the highest sensitivity (77.1%) and combined with conventional sputum smear and culture significantly increased the sensitivity further to 84.2%. In conclusion, Xpert‐MTB/RIF is a sensitive, rapid, and reliable method for TB detection in sputum samples, and other diagnostic methods including culture are still of great clinical values for improving the sensitivity of MTB diagnosis. The sensitivity of CapitalBio TB/NTM kit in diagnosing NTM is still insufficient in clinical practice.

## Graphical abstract

Correct diagnosis of tuberculosis is crucial for controlling its spread. This study evaluated five diagnostic methods for *Mycobacterium tuberculosis* (MTB) and nontuberculous mycobacteria (NTM) in 3012 sputum samples. Xpert‐MTB/RIF showed the highest sensitivity for MTB (79.6%), while combining CapitalBio TB/NTM kit and culture increased sensitivity to 88.4%. For mycobacteria, the CapitalBio TB/NTM kit detection test has the highest sensitivity (77.1%), and the conventional sputum smear and the sandwich cup sputum smear had no significant difference in sensitivity.

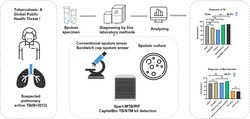

## 1 Introduction

Tuberculosis (TB) is a global public health threat as an infectious disease. According to Global Tuberculosis Report 2021, an estimated10 million people were infected with TB and 1.5 million people died from TB in 2020. TB is the 13th leading cause of death and the second leading infectious killer after COVID‐19 [1].

Effective blocking of TB transmission relies on timely diagnosis and treatment. Sputum smear microscopy remains a primary diagnostic technique for evaluating individuals with signs and symptoms of pulmonary TB in many high‐burden areas [2]. Although inexpensive and easy to implement, it has low sensitivity with a detection limit of 5000–10 000 bacilli per milliliter of sputum [3]. Furthermore, sputum smear microscopy cannot distinguish between MTB and nontuberculous mycobacteria (NTM). A previous study suggested that concentration of bacilli through small‐membrane filter can substantially improve the sensitivity of direct microscopy by sandwich cup sputum smear, which is a new method for safe and rapid acid‐fast bacilli (AFB) concentration and detection [4]. A previous study demonstrated that the sensitivity of concentrated smear microscopy was improved when using positive culture as the gold standard (83% vs.71%) [5]. Culture is the gold standard method used as a bacteriologic confirmation of TB cases; however, due to biosafety concerns, costs, infrastructure requirements, and long time needed to produce results, culture is not utilized as the primary diagnosis in many high‐burden countries [6]. In addition to the culture method, molecular biological tests serve as another method for laboratory pathogen confirmation. The Xpert‐MTB/RIF test is a cartridge‐based real‐time PCR assay for simultaneous detection of MTB and rifampicin resistance from sputum specimens in less than 2 h, which was recommended for TB diagnosis by the WHO in 2010 [6]. Another commonly used clinical technology is real‐time fluorescence PCR, which primarily targets IS6110 for differential diagnosis of *Mycobacterium tuberculosis* and NTM, characterized by high sensitivity and fast detection rate [7]. In recent years, with the development of technology, digital PCR technology has also offered a sight in the diagnosis of TB, which is a new type of PCR technology between high‐throughput sequencing and real‐time PCR technology. It is mainly applied to absolute quantification of *M. tuberculosis* determination with high precision. However, its high cost has limited its clinical use [8].

Since the effectiveness of the various assays was not consistent and satisfactory, this study evaluated five laboratory tests, including conventional sputum smear microscopy, sandwich cup sputum smear microscopy, sputum culture, Xpert‐MTB/RIF, and CapitalBio TB/NTM kit detection test and their combinations, to enhance TB diagnosis performance under the routine bacteriology laboratory conditions.

## 2 Study Design, Materials, and Methods

We screened all patients admitted to Taizhou Enze Hospital (3 February 2019 to 11 June 2019), Ningbo No. 2 Hospital (6 July 2019 to 24 October 2019), and Hangzhou Red Cross Hospital (16 March 2019 to 7 July 2019) with suspected active pulmonary TB with supportive clinical symptoms and chest imaging evidence. Sputum samples were collected from each enrolled patient and presented for various TB tests.

All sputum samples were subjected to three kinds of laboratory methods, including AFB by sputum smear microscopy, *Mycobacterium* culture, and molecular biological tests. Firstly, the sputum samples were digested with N‐acetyl‐L‐cysteine‐NaOH in sodium citrate (1.5% final NaOH concentration), vortexed for 30 s, and incubated for 15 min at room temperature (approximately 25°C). The processed samples were subsequently inoculated onto Löwenstein–Jensen medium (Baso, Zhuhai, Guangdong Province, China). After culturing the isolates, a colloidal gold assay (Genesis, Hangzhou, Zhejiang Province, China) was conducted to detect MPT64 antigen according to routine procedures for differentiating MTB from NTM.

In addition to conventional sputum smear microscopy, a sandwich cup sputum smear assay (Tianqi, Huaihua, Hunan Province, China) was used in this study comparatively for all sputum samples. Sputum samples were treated with a lysis buffer provided by the assay. The digested sputum was filtered through a shadowless membrane sandwich cup and the acid bacilli in the samples to be intercepted and aggregated on the nanosilicon media microporous filter membrane sheet. The membrane was subsequently stained on the membrane sheet and directly used for microscopic examination. At least 300 microscopic fields per negative slide and 100 fields per positive slide were observed.

Both Xpert‐MTB/RIF (Cepheid Inc., California, United States) and CapitalBio TB/NTM (CapitalBio Technology Inc., Beijing, China) kit detection test were used as molecular biological methods for the detection of TB and NTM. All sputum samples were subjected to the Xpert‐MTB/RIF Ultra cartridge (Cepheid Inc., California, United States) and loaded into the automated instrument according to the manufacturer's instructions. CapitalBio TB/NTM kit detection test extracted nucleic acids from digested sputum samples using *Mycobacterium* nucleic acid test kit, which utilizes dual real‐time fluorescent PCR and TaqMan probe technology to differentiate between MTB and NTM [9]. The interpretation of the results of this method is mainly based on the CT values of the samples, with positive FAM channel for TB, negative FAM channel and positive VIC channel for NTM, and negative FAM channel and negative VIC channel for negative results.

## 3 Statistical Analysis and Diagnostic Criteria

**FIGURE 1:**
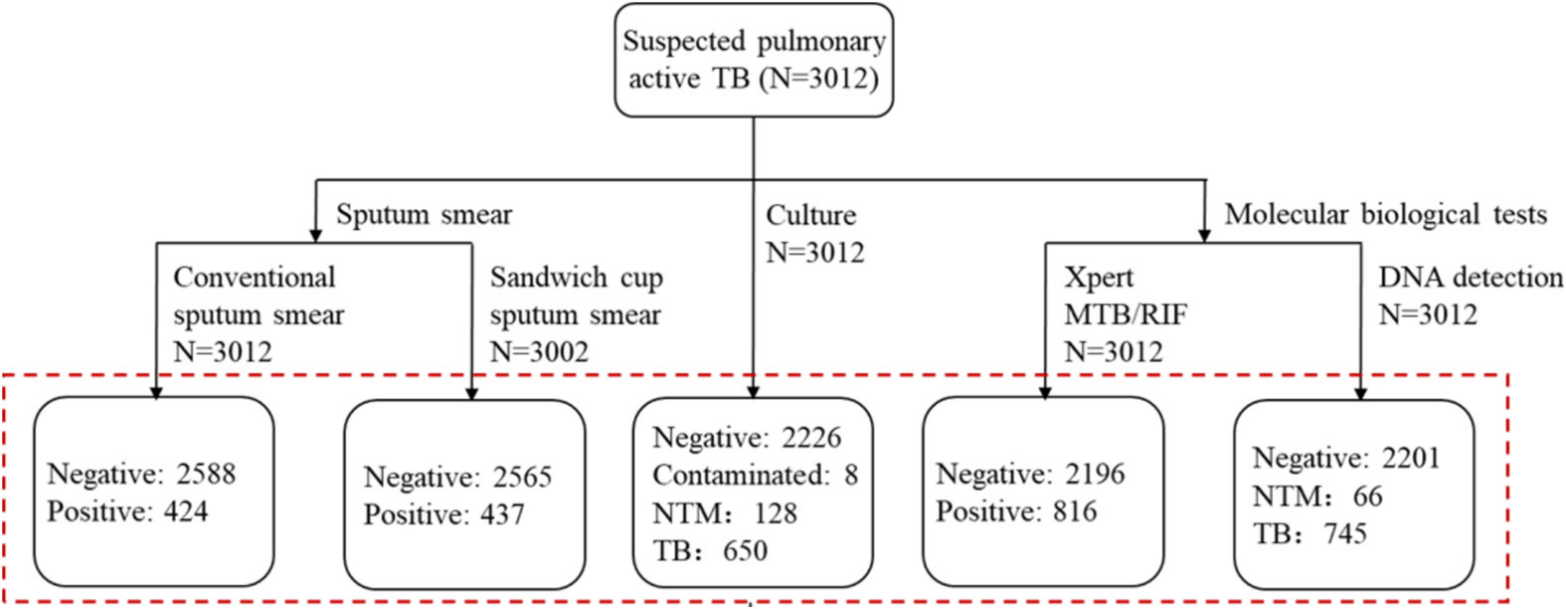
Workflow of diagnosis of 3012 suspected pulmonary active tuberculosis patients.

For the diagnosis of *M. tuberculosis* , the gold standard for positivity is either positive results of culture method combined with MPT64 antigen detection, Xpert‐MTB/RIF, or CapitalBio TB/NTM kit detection test. Culture positive is the gold standard for diagnosis of NTM, and either positive of the culture method or CapitalBio TB/NTM kit detection test is the gold standard for the mycobacteria diagnosis in our study. In total, there were 10 samples for which culture, Xpert‐MTB/RIF, and CapitalBio TB/NTM kit detection test showed conflicting pathogen results concerning TB and NTM, potentially indicating a mixed infection (Figure 1 and Table S1). In analyzing the diagnostic efficacy of MTB or NTM, we excluded the data of the above 10 samples.

Sensitivity and specificity for different methods were calculated with the range of 95% confidence intervals. All data analyses were conducted using SPSS Statistics 27 software. McNemar's chi‐square test was used to compare the positive rate and sensitivity of different methods. A *p* value of < 0.05 was considered statistically significant.

## 4 Results

Totally, 3012 sputum samples from patients suspected with pulmonary TB from February to October 2019 in three TB designated hospitals were included in this study. All samples were conducted four TB testing including culture, conventional sputum smear, Xpert‐MTB/RIF, and CapitalBio TB/NTM kit detection test. Additionally, 3002 samples were conducted sandwich cup sputum smear due to insufficient specimen. The number of specimens diagnosed as TB, NTM, and negative were 650, 128, and 2226, respectively, for culture method and 745, 66, and 2201, respectively, for CapitalBio TB/NTM kit detection test. The number of specimens detected as MTB positive for conventional sputum smear, sandwich cup sputum smear, and X‐pert‐MTB/RIF method was 424, 437, and 816, respectively, while that detected as negative was 2588, 2565, and 2196, respectively (Figure 1).

### 4.1 Diagnostic Performance for MTB

In our experiments, three methods were employed; CapitalBio TB/NTM kit detection test, Xpert‐MTB/RIF, and culture were used for the diagnosis of MTB, while the other method cannot distinguish between MTB and NTM. For single method detection, the sensitivity of the Xpert‐MTB/RIF reached 79.6% (0.770–0.819), which was higher than that of CapitalBio TB/NTM kit detection test and the culture method (*p* < 0.01), which were 72.3% (0.695–0.750) and 63.6% (0.606–0.665), respectively (Table 1). The combination of the CapitalBio TB/NTM kit detection test and the culture methods achieved the highest sensitivity 88.4% (0.863–0.902) among two‐test combinations, which is higher than any single test (*p* < 0.01). The combination of Xpert‐MTB/RIF and the culture demonstrated a sensitivity of 85.5% (0.832–0.875) (Table 1) (*p* < 0.01).

**TABLE 1:** Diagnostic performance of each single test and combined tests for TB and NTM.

Test/test combination	Target	Sensitivity	Specificity
CapitalBio TB/NTM kit detection test	TB	72.3% (0.695–0.750)^a^	100.0%
Sputum culture	TB	63.6% (0.606–0.665)^b^	98.9% (0.982–0.992)
Xpert‐MTB/RIF	TB	79.6% (0.770–0.819)	100.0%
CapitalBio TB/NTM kit detection test	NTM	45.7% (0.267–0.430)	100.0%
Culture	NTM	85.7% (0.790–0.906)	100.0%
CapitalBio TB/NTM kit detection test + sputum culture	TB	88.4% (0.863–0.902)^c^	100.0%
Xpert‐MTB/RIF + sputum culture	TB	85.5% (0.832–0.875^d^	98.8% (0.982–0.992)

^a^ CapitalBio TB/NTM kit detection test versus Xpert‐MTB/RIF, *p* < 0.01.

^b^ Sputum culture versus Xpert‐MTB/RIF, *p* < 0.01.

^c^ CapitalBio TB/NTM kit detection test + culture versus CapitalBio TB/NTM kit detection test, *p* < 0.01.

^d^ Xpert‐MTB/RIF + sputum culture versus Xpert‐MTB/RIF, *p* < 0.01.

### 4.2 Diagnostic Performance for NTM

Only the CapitalBio TB/NTM kit detection test and the culture method can distinguish NTM from mycobacteria‐positive specimens. The sensitivity of the culture method was 85.7% (0.790–0.906) for diagnosing NTM, which was significantly higher than that of CapitalBio TB/NTM kit detection test (*p* < 0.01), which was 45.7% (0.377–0.540) (Table 1).

### 4.3 Diagnostic Performance for Mycobacteria

Totally, four assays including CapitalBio TB/NTM kit detection test, conventional sputum smear, sandwich cup sputum smear, and culture were used for the diagnosis of mycobacteria. For single tests, the sensitivity of CapitalBio TB/NTM kit detection test was 77.1% (0.744–0.795), ranking the first. The sensitivities of sputum culture, sandwich cup sputum smear, and conventional sputum smear were 74.0% (0.713–0.766), 35.2% (0.324–0.381), and 36.9% (0.324–0.381), respectively. Comparatively, the CapitalBio TB/NTM kit detection test and the culture had no significant difference in sensitivity (*p* = 0.172), nor had the conventional sputum smear and the sandwich cup sputum smear (*p* = 0.415). When we combined the conventional sputum smear with culture, the sensitivity reached 76.9% (0.742–0.793), which was higher than the culture (*p* < 0.01). The combination of the sputum smear, sputum culture, and CapitalBio TB/NTM test had the highest sensitivity of 84.2% (0.819–0.863), which is higher than that of any single test (*p* < 0.01) (Table 2).

**TABLE 2:** Diagnostic performance of each single test and combined tests for mycobacteria.

Test/test combination	Target	Sensitivity	Specificity
CapitalBio TB/NTM kit detection test	Mycobacteria	77.1% (0.744–0.795)^a^	100.0%
Sputum culture	Mycobacteria	74.0% (0.713–0.766)	99.6% (0.992–0.998)
Conventional sputum smear	Mycobacteria	36.9% (0.341–0.399)^b^	98.2% (0.955–0.971)
Sandwich cup sputum smear	Mycobacteria	35.2% (0.324–0.381)	96.4% (0.975–0.987)
Conventional sputum smear + sputum culture	Mycobacteria	76.9% (0.742–0.793)^c^	97.8% (0.971–0.984)
Conventional sputum smear + sputum culture + CapitalBio TB/NTM kit detection test	Mycobacteria	84.2% (0.819–0.863)^d^	98.2% (0.975–0.987)

^a^ CapitalBio TB/NTM kit detection test versus sputum culture, *p* = 0.172.

^b^ Conventional sputum smear versus sandwich cup sputum smear, *p* = 0.415.

^c^ Conventional sputum smear + sputum culture versus sputum culture, *p* < 0.01.

^d^ Conventional sputum smear + sputum culture + CapitalBio TB/NTM kit detection test versus CapitalBio TB/NTM kit detection test, *p* < 0.01.

## 5 Discussion

TB is a chronic infectious disease caused by infection with MTB, which exhibits latency and typically and develops only when the body's immune system decreases. Furthermore, MTB can invade several organs including the invasion of lung tissue and transmission through close contact is common. Therefore, early, rapid, and accurate diagnosis of TB is very important for the prevention, control, and treatment of the disease. In this study, 3012 patients were identified and diagnosed with *M. tuberculosis* using five different bacteriological and molecular biological examination methods. Studies comparing TB diagnostic methods are not uncommon, but those that include such a large number of samples are of greater significance.

In our study, the Xpert‐MTB/RIF assay demonstrated the highest sensitivity (79.6%) among all single tests for diagnosing *M. tuberculosis* , consistent with findings by Huang and Afsar [10, 11]. A previous meta‐analysis of 22 studies involving 8998 participants indicated the pooled sensitivity of Xpert‐MTB/RIF was 89% [95% credible interval (CI) 85%–92%] with a pooled specificity of 99% (95% CI 98%–99%) [12]. In addition, another meta‐analysis demonstrated that in 25 previous studies, the pooled sensitivity and specificity of the diagnosis of TB were 89% and 98% for Xpert‐MTB/RIF, respectively [13]. The sensitivity observed in this study (79.6%) is slightly lower than reported in the aforementioned metastudies, as we found that the overall positive detection rate of sputum was slightly improved due to the simultaneous use of multiple methods for TB detection. When we combined the two molecular assays, CapitalBio TB/NTM kit detection test and Xpert‐MTB/RIF, respectively, with the culture method, the sensitivities of the combined assay were significantly higher than those of the single assay, which suggested that the combined use of multiple tests can improve diagnostic efficacy [10]. The Xpert MTB/RIF test is highly sensitive and rapid and yields easily interpretable results, but it is costly due to the requirement for specialized equipment [14]. These findings highlight the importance of employing comprehensive diagnostic strategies in clinical settings to optimize TB detection and management.

For the diagnosis of both TB and NTM, we found that the CapitalBio TB/NTM kit detection test has the highest sensitivity (77.10%) among the single assays. This finding is consistent with previous studies; the PCR assay is more sensitive and accurate than the traditional smear and culture methods and can detect *M. tuberculosis* even in the presence of negative sputum smears [9, 15]. A previous study showed that based on culture as the gold reference, the sensitivity of CapitalBio TB/NTM kit detection test was 83.0% (80.8%–85.0%) for TB and was 53.1% for NTM [16], which was more sensitive than AFB smear for both TB and NTM diagnoses. In another study, the CapitalBio TB/NTM kit detection test had a higher sensitivity than Xpert MTB/RIF in the diagnosis of TB‐associated diseases such as tuberculous meningitis and spinal TB [17]. Given that cultures take a long time to produce results, PCR assay allows for rapid early diagnosis and is being promoted in the clinical setting. These findings underscore the utility of PCR‐based assays, such as the CapitalBio TB/NTM kit, in enhancing the sensitivity of TB and NTM diagnosis, particularly in situations where rapid detection is crucial for effective patient management.

For the detection of NTM, the CapitalBio TB/NTM kit detection test exhibited the sensitivity of only 45.7% (0.267–0.430), which was lower than that reported in other studies. In a previous study, the sensitivity of the CapitalBio TB/NTM kit detection test was reported to be 53.1% [16]. The reason for the low sensitivity in our study may be attributed to differences in specimen types. We tested only sputum specimens, while the previous study utilized alveolar lavage fluid specimens. Another potential reason for low sensitivity may be that our samples were collected from one province, which exhibits regional differences compared to the areas discussed in the previous study [18]. Furthermore, variations in laboratory conditions may also influence the identification result [19]. Consequently, achieving a more rapid and accurate diagnosis of NTM requires further research efforts to refine and optimize detection methodologies.

The sandwich cup sputum smear is mainly characterized by centrifugal filtration and abundant shaking to accumulate all liquefied completely sputum on a single membrane, which is then stained with acid for microscopic observation, and its minimum detection limit has been reported to be lower than that of the conventional method [20]. Previous studies have reported that the sensitivities of concentrated smear microscopy ranged from 63% to 89% and higher than those the of direct smear microscopy, 50%–57% [21]. However, the diagnostic sensitivity in our study was not superior to that of the conventional smear method. Although these methods are simple, safe, and relatively inexpensive to perform [22], they all exhibit low and unsatisfactory sensitivity for diagnosing mycobacteria.

There are still some shortcomings in our study. The data were collected only from Zhejiang Province and all the specimens were conducted in only one laboratory; that laboratory’s operating habits and conditions may have affected the results of testing methods. Secondly, all specimens in our study were sputum, and the diagnostic performance and efficacy of the methods were not evaluated in other types of samples. Thirdly, because the gold standard for diagnosis was derived from the testing methods themselves, it is not possible to conduct a specificity comparison between these methods in this study. In terms of specificity, traditional culture and smear methods are generally more specific than molecular methods and it may be related to nucleic acid contamination. Except for contaminations, fragments of dead bacteria in the sample cannot be cultured, but its nucleic acid testing may still be positive. In this study, 10 out of 3012 specimens exhibited conflicting results among CapitalBio TB/NTM kit detection test, Xpert‐MTB/RIF, and culture method. Most inconsistencies occurred when the culture method detected NTM but TB positive in nucleic acid assay. This may have been caused by contaminations or coinfected with both TB and NTM.

## 6 Conclusion

In summary, although Xpert‐MTB/RIF is a sensitive, rapid, and reliable method for TB detection in sputum samples, the combination use of the CapitalBio TB/NTM kit detection test and culture is valuable for enhancing the sensitivity of TB diagnosis. The sensitivity of the CapitalBio TB/NTM kit detection test for detecting NTM from sputum is insufficient. Currently, except for *Mycobacterium* culture, there is still a lack of powerful tools that can diagnose both NTM and TB simultaneously from sputum samples.

## Author Contributions

Yelei Zhu, Zhengwei Liu, Mingwu Zhang, Jiazhen Chen, and Beibei Wu contributed to conception and design of the study. Qian Wu, Yelei Zhu, and Yu Zhang performed the data extraction and statistical analysis. Qian Wu wrote the main manuscripts. Jiazhen Chen and Beibei Wu administered and supervised the project. All authors reviewed and approved the final version of the manuscript.

## Ethics Statement

This study was approved by ethical committees in of Zhejiang Provincial Center for Disease Control and Prevention (No. 20180905) and we had already obtained written informed consent from all study subjects.

## Conflicts of Interest

The authors declare no conflicts of interest.

## Supporting information


**Table S1** Ten samples with conflicted results among Xpert‐MTB/RIF, CapitalBio TB/NTM kit detection test, and culture.

## Data Availability

The data that support the findings of this study are available from the corresponding author upon reasonable request.

## References

[1] J. Chakaya , M. Khan , F. Ntoumi , et al., “Global Tuberculosis Report 2020 – Reflections on the Global TB Burden, Treatment and Prevention Efforts,” International Journal of Infectious Diseases 113 (2021): S7–S12. 33716195 10.1016/j.ijid.2021.02.107 PMC8433257

[2] H. S. Cox , S. Mbhele , N. Mohess , et al., “Impact of Xpert MTB/RIF for TB Diagnosis in a Primary Care Clinic With High TB and HIV Prevalence in South Africa: A Pragmatic Randomised Trial,” PLoS Medicine 11, no. 11 (2014): e1001760. 25423041 10.1371/journal.pmed.1001760 PMC4244039

[3] P. Quinco , S. Buhrer‐Sekula , W. Brandao , et al., “Increased Sensitivity in Diagnosis of Tuberculosis in HIV‐Positive Patients Through the Small‐Membrane‐Filter Method of Microscopy,” Journal of Clinical Microbiology 51, no. 9 (2013): 2921–2925. 23804389 10.1128/JCM.00683-13 PMC3754651

[4] D. Helb , M. Jones , E. Story , et al., “Rapid Detection of Mycobacterium tuberculosis and Rifampin Resistance by Use of On‐Demand, Near‐Patient Technology,” Journal of Clinical Microbiology 48, no. 1 (2010): 229–237, 10.1128/JCM.01463-09. 19864480 PMC2812290

[5] T. J. Chandra , R. Selvaraj , and Y. V. Sharma , “Same‐Day Sputum Smear Microscopy for the Diagnosis of Pulmonary Tuberculosis: Direct vs. Concentrated Smear,” International Journal of Tuberculosis and Lung Disease 20, no. 2 (2016): 247. 10.5588/ijtld.15.0566 26792479

[6] WHO Consolidated Guidelines on Tuberculosis: Module 3: Diagnosis – Rapid Diagnostics for Tuberculosis Detection (World Health Organization, 2020). 33999549

[7] H. Y. Wang , J. J. Lu , C. Y. Chang , et al., “Development of a High Sensitivity TaqMan‐Based PCR Assay for the Specific Detection of Mycobacterium tuberculosis Complex in Both Pulmonary and Extrapulmonary Specimens,” Scientific Reports 9, no. 1 (2019): 113. 30643154 10.1038/s41598-018-33804-1 PMC6331544

[8] R. Nyaruaba , C. Mwaliko , K. K. Kering , and H. Wei , “Droplet Digital PCR Applications in the Tuberculosis World,” Tuberculosis (Edinburgh, Scotland) 117 (2019): 85–92, 10.1016/j.tube.2019.07.001. 31378274

[9] H. Zheng , F. Zhong , G. Yu , and Y. Shen , “Comparison of the Diagnostic Efficacy of the Capitalbio Mycobacterium Real‐Time Polymerase Chain Reaction Detection Test and Xpert mtb/rif in Smear‐Negative Pulmonary Tuberculosis,” European Journal of Clinical Microbiology & Infectious Diseases 40, no. 5 (2021): 969–977. 33242168 10.1007/s10096-020-04113-1

[10] F. Huang , L. Dang , H. Sun , H. Yang , and X. Wu , “A Study of the Value of Three Molecular Diagnostic Techniques in the Diagnosis of Tuberculosis,” Chinese Journal of Tuberculosis and Respiratory Diseases 38, no. 9 (2015): 680–685. 26703773

[11] I. Afsar , M. Gunes , M. Er , and A. G. Sener , “Comparison of Culture, Microscopic Smear and Molecular Methods in Diagnosis of Tuberculosis,” Revista Espanola de Quimioterapia 31, no. 5 (2018): 435–438. 30229645 PMC6194869

[12] K. R. Steingart , I. Schiller , D. J. Horne , M. Pai , C. C. Boehme , and N. Dendukuri , “Xpert® MTB/RIF Assay for Pulmonary Tuberculosis and Rifampicin Resistance in Adults,” Cochrane Database of Systematic Reviews 2014, no. 1 (2014): CD009593, 10.1002/14651858.CD009593.pub3. 24448973 PMC4470349

[13] L. Yan , H. Xiao , and Q. Zhang , “Systematic Review: Comparison of Xpert MTB/RIF, LAMP and SAT Methods for the Diagnosis of Pulmonary Tuberculosis,” Tuberculosis 96 (2016): 75–86. 26786658 10.1016/j.tube.2015.11.005

[14] C. C. Boehme , M. P. Nicol , P. Nabeta , et al., “Feasibility, Diagnostic Accuracy, and Effectiveness of Decentralised Use of the Xpert mtb/rif Test for Diagnosis of Tuberculosis and Multidrug Resistance: A Multicentre Implementation Study,” Lancet 9776 (2011): 377. 10.1016/S0140-6736(11)60438-8 PMC3085933 21507477

[15] N. Rakotosamimanana , M. S. Rabodoarivelo , J. C. Palomino , A. Martin , and V. R. Razanamparany , “Exploring Tuberculosis by Molecular Tests on PCR‐Flurescence Peobe Assay Isolated From Smear Microscopy Slides,” International Journal of Infectious Diseases IJID Official Publication of the International Society for Infectious Diseases 56, no. Complete (2016): 248–252. 27979786 10.1016/j.ijid.2016.12.005

[16] Y. Shen , L. Fang , X. Xu , B. Ye , and G. Yu , “Capitalbio Mycobacterium Real‐Time Polymerase Chain Reaction Detection Test: Rapid Diagnosis of Mycobacterium tuberculosis and Nontuberculous Mycobacterial Infection,” International Journal of Infectious Diseases 98 (2020): 1–5. 32553719 10.1016/j.ijid.2020.06.042

[17] L. Sun , L. Yao , G. Fu , L. Lin , E. Zhu , and J. Huang , “A Comparison of the Accuracy of the CapitalBio Mycobacterium Real‐Time Polymerase Chain Reaction and the Xpert MTB/RIF Assay for the Diagnosis of Tuberculous Meningitis,” International Journal of Infectious Diseases: IJID: Official Publication of the International Society for Infectious Diseases 104 (2021): 92–96. 33352329 10.1016/j.ijid.2020.12.044

[18] E. E. Mcgrath , J. Mccabe , and P. B. Anderson , “Guidelines on the Diagnosis and Treatment of Pulmonary Non‐Tuberculous Mycobacteria Infection,” International Journal of Clinical Practice 62, no. 12 (2010): 1947–1955. 10.1111/j.1742-1241.2008.01891.x 19166441

[19] L. Yao , C. Bu , J. Zhang , and D. Zhang , “The Value of Histopathology Combined With CapitalBio Mycobacterium Real‐Time Polymerase Chain Reaction Test for Diagnosing Spinal Tuberculosis,” Frontiers in Medicine 10 (2023): 1173368. 37425306 10.3389/fmed.2023.1173368 PMC10326313

[20] J. Peng , L. Wen‐En , L. Hong‐Ling , L. Qing‐Xia , and P. Wan‐Chan , “Evaluation of Our Self‐Designed Nanometer Silicon Membrane Sandwich Cup System for Diagnosing Tuberculosis,” Clinical Respiratory Journal 10, no. 5 (2016): 647–652, 10.1111/crj.12273. 25620164

[21] M. K. M. Uddin , M. R. Chowdhury , S. Ahmed , M. T. Rahman , and S. Banu , “Comparison of Direct Versus Concentrated Smear Microscopy in Detection of Pulmonary Tuberculosis,” BMC Research Notes 6(2013‐07‐25), no. 1 (2013): 1–6. 23885922 10.1186/1756-0500-6-291 PMC3733684

[22] H. Y. Wang , H. Kim , S. Kim , H. Bang , D. K. Kim , and H. Lee , “Evaluation of PCR‐Reverse Blot Hybridization Assay for the Differentiation and Identification of Mycobacterium Species in Liquid Cultures,” Journal of Applied Microbiology 118, no. 1 (2015): 142–151, 10.1111/jam.12670. 25346202

