# Supplementary material for: Evaluation and Comparison of Laboratory Methods in Diagnosing Mycobacterium tuberculosis and Nontuberculous Mycobacteria in 3012 Sputum Samples
Source: Clin Respir J. 2025 Mar 14;19(3):e70071. doi: 10.1111/crj.70071 (PMC11908973; doi:10.1111/crj.70071)
Supplement: Supplementary file 1 — Table S1 Ten samples with conflicted results among Xpert‐MTB/RIF, CapitalBio TB/NTM kit detection test, and culture. [file CRJ-19-e70071-s001.pdf]

Supplemental Material

Table S1. Ten samples with conflicted results among Xpert-MTB/RIF, CapitalBio TB/NTM kit detection test and culture.

No.	Xpert-MTB/RIF	CapitalBio TB/NTM kit detection test	Culture
P0148	TB	TB	NTM
P0156	(-)	TB	NTM
P0163	TB	TB	NTM
P0231	(-)	TB	NTM
P0301	TB	(-)	NTM
P0968	TB	NTM	TB
P1002	(-)	TB	NTM
P1202	(-)	TB	NTM
P1282	(-)	TB	NTM
P1392	TB	NTM	TB
